# Supplementary material for: Exploring user and stakeholder perspectives from South Africa and Uganda to refine microarray patch development for HIV PrEP delivery and as a multipurpose prevention technology
Source: PLoS One. 2023 Aug 31;18(8):e0290568. doi: 10.1371/journal.pone.0290568 (PMC10470907; doi:10.1371/journal.pone.0290568)
Supplement: Attachment — Submitted filename: Response.docx [file pone.0290568.s001.docx]

January 2023

Editorial Board

*PLOS One*

RE: PONE-D-22-31097

Greetings,

Thank you for your review of our submission “*Exploring user and stakeholder perspectives from South Africa and Uganda to refine microarray patch development for HIV PrEP delivery and as a multipurpose prevention technology*.” We are pleased to submit this cover/rebuttal letter along with a revised manuscript and associated files to *PLOS One* for consideration.

**Review comments and rebuttal**

**1.** Please ensure that your manuscript meets PLOS ONE's style requirements, including those for file naming. The PLOS ONE style templates can be found at 
<https://journals.plos.org/plosone/s/file?id=wjVg/PLOSOne_formatting_sample_main_body.pdf> and 
<https://journals.plos.org/plosone/s/file?id=ba62/PLOSOne_formatting_sample_title_authors_affiliations.pdf>

*We have made minor formatting changes to comply with PLOS ONE’s style requirements. Apologies that we missed these the first time.*

**2.** **Thank you for stating the following in the Acknowledgments Section of your manuscript:** “These assessments and this manuscript were made possible by the generous support of the American people through the United States Agency for International Development (USAID) through the United States President’s Emergency Plan for AIDS Relief (PEPFAR), under the terms of Cooperative Agreement #AID-OAA-A-17-00015.”

We note that you have provided funding information that is not currently declared in your Funding Statement. However, funding information should not appear in the Acknowledgments section or other areas of your manuscript. We will only publish funding information present in the Funding Statement section of the online submission form. Please remove any funding-related text from the manuscript and let us know how you would like to update your Funding Statement. Currently, your Funding Statement reads as follows:

“-Initial of author who received the award: CJ
-Grant number: AID-OAA-A-17-00015.
-Funder name: US Agency for International Development (USAID) 
-Website: <https://www.usaid.gov/>

NO. The funder had no role in study design, data collection and analysis, decision to publish, or preparation of the manuscript.”

Please include your amended statements within your cover letter; we will change the online submission form on your behalf.

*Thank you for pointing this out. We have removed the funding language from the manuscript. The Funding Statement in the online submission is correct as stated above.*

**3.** **PLOS requires an ORCID iD for the corresponding author in Editorial Manager**.

*We have connected the submission with my ORCID ID, which is:* [*0000-0001-6161-7213*](https://orcid.org/0000-0001-6161-7213)

**4.** **We note that you have included the phrase “data not reported here” in your manuscript**. Unfortunately, this does not meet our data sharing requirements. PLOS does not permit references to inaccessible data. We require that authors provide all relevant data within the paper, Supporting Information files, or in an acceptable, public repository. Please add a citation to support this phrase or upload the data that corresponds with these findings to a stable repository (such as Figshare or Dryad) and provide and URLs, DOIs, or accession numbers that may be used to access these data. Or, if the data are not a core part of the research being presented in your study, we ask that you remove the phrase that refers to these data.

*Thank you for catching this statement. We have removed the phrase “data not reported here” from the manuscript. Results for all core parts of the study are included in the manuscript.*

**5.** **Please review your reference list to ensure that it is complete and correct**. If you have cited papers that have been retracted, please include the rationale for doing so in the manuscript text, or remove these references and replace them with relevant current references. Any changes to the reference list should be mentioned in the rebuttal letter that accompanies your revised manuscript. If you need to cite a retracted article, indicate the article’s retracted status in the References list and also include a citation and full reference for the retraction notice.

*We checked the references and have made minor revisions as follows:*

*In the Introduction and Materials and methods sections:*

- *We removed a reference that described the “monthly oral pill” that was being developed as an additional HIV PrEP product since the developer halted further development of this ART for HIV PrEP [former reference 13].*
- *In the same sentence, we added text about new HIV prevention technologies in development and a reference to the MATRIX website [new reference 13].*
- *We added a reference regarding the rationale for multipurpose prevention [new reference 16].*
- *We added references describing development of MAPs for various health indications [new references 33 and 34]*
- *We updated cross-references to reflect current numbers.*

*In the References section:*

- *We added the reference text for the new references noted above—13, 16, 33, 34—and removed other text as needed*
- *We corrected a link that was not functional in an existing reference [current reference 32].*
  *PATH. The PATH Center of Excellence for Microarray Patch Technology: Advancing the Microarray Patch Delivery Technology Platform for High-Priority Global Health Needs. Seattle: PATH; 2019. Available from:* *[https://media.path.org/documents/MAPs-fs-Jan2020.pdf](https://media.path.org/documents/MAPs-fs-Jan2020.pdf?_gl=1*epjq98*_ga*MzI0NTc5MDUyLjE2Mzk3NzE3ODM.*_ga_YBSE7ZKDQM*MTY3NDg2MzE3OC4xNy4xLjE2NzQ4NjMyMTAuMC4wLjA).*

**6.** **Human subjects were used in this study. Please submit IRB approval/s with the manuscript.**

*Apologies for misunderstanding the prompt. As noted at the end of the Materials and methods section of the manuscript, in South Africa, ethics approval was obtained from the University of Witwatersrand Human Research Ethics Committee (Medical) (Clearance Certificate No. M190721). In Uganda, ethics approved was provided by the Makerere University School of Medicine Research Ethics Committee and the Uganda National Council for Science and Technology (UNCST SS5090 in Uganda. We have submitted the three* *approval letters as Supporting information (S1, S2, and S3) and corrected the submission coversheet to indicate that these studies included Human Subjects ethics review.*

Authors declare no competing interests, and the authors confirm that the manuscript, or any related manuscript, is not currently under consideration or accepted elsewhere. To provide full transparency, we wish to inform you that preliminary findings from these assessments were presented at the HIV Research for Prevention Conference in South Africa in January 2021.

Please contact me if you have any additional questions about this manuscript.

Best wishes,

Maggie Kilbourne-Brook

Corresponding author

Senior Program Officer, PATH

[mkilbou@path.org](mailto:mkilbou@path.org)
