# Supplementary material for: Exploring user and stakeholder perspectives from South Africa and Uganda to refine microarray patch development for HIV PrEP delivery and as a multipurpose prevention technology
Source: PLoS One. 2023 Aug 31;18(8):e0290568. doi: 10.1371/journal.pone.0290568 (PMC10470907; doi:10.1371/journal.pone.0290568)
Supplement: Attachment — Submitted filename: Response to Reviewers.docx [file pone.0290568.s002.docx]

**RESPONSE TO REVIEWERS**

***Review comments*** *(black font)* *and* ***response*** *(indented, blue)*

**REVIEWER #1:**

**Abstract**

Line 25-26: It is not clear to which group the male partners were. "Participants included adolescent girls and young women, female sex workers and male partners of these women as well as men who have sex with men."

**Response**: Thank you for this comment. We have revised the abstract per your suggestion.

**Methods**

Line 123: Review the epidemiological research design. "We used a cross sectional qualitative research design or approach..."

**Response**: We have revised this sentence per your suggestion to tighten the description of the research design, and to respond to a question from Reviewer #2. Thank you. This sentence now reads:

Line 196-197, Table 2 on page 12: Inconsistences in the listed participant groups as stated in abstract and methods sections. AGYW (Community), AGYW (University)

**Response**: Thank you for pointing out this discrepancy. We revised the abstract to clarify these groups and make the text consistent. This portion of the abstract now reads:

Line 26 and Table 2 on page 12: What was the criterion of including the men participants?

**Response**: Participants recruited for this assessment were aged 18–40 years, and included young women and men from key populations and vulnerable groups at risk of HIV and/or unintended pregnancy. We recruited men both for their potential role as “end-users” of an HIV PrEP MAP and also as male partners who influence women’s choice and ability to use HIV prevention strategies and contraception. We added a Table 2 footnote to clarify.

**Results**

Page 11: Demographic information to introduce the participants is sometimes helpful. Insert that paragraph.

**Response**: We added detail at the beginning of the Results section to clarify the demographic profile of study participants. Please see the image below to see how this section now reads:

Page 11 and Table 2: Table 2 seems to fit better in data management and analysis section.

**Response**: Thank you for this comment. We believe Table 2 belongs in the **Results** section because it provides an overview of the research activities that were completed, by activity and by site. The table title was confusing, so we have revised the table title to better reflect the content.

**REVIEWER #2:**

**Abstract**

Add a line about data analysis in the abstract.

**Response**: We added the sentences to the abstract to address this comment:

Line 35: acceptable?

**Response**: Thank you for this suggestion that the word “acceptable” might be a better choice than “desirable”. The response among the potential user groups and stakeholders was stronger than just acceptable. Self- administration is perceived as a *valued* characteristic of a MAP product, so we have used this word. The sentence now reads:

**Introduction**

Line 75: spell out and define MPTs.

**Response**: Thank you for catching this. We’ve spelled out multipurpose prevention technologies (MPT), as this is the first use of this abbreviation in the manuscript.

**Methods**

Line 175: specify the local languages.

Setting of activities: Describe the settings of mock exercises, IDIs, and FGDs where were they done? The FGDs and mock exercises were made up of how many people? How were they stratified? How long did they (IDIs, FGDs, and Mock use sessions) take?

**Response**: Thank you for these comments/recommended edits. We revised the text to address these points:

Likert-style scale: The authors mentioned that a survey (Likert Scale) was done in Uganda to assess mock-use exercises. However, there is no information on how that data was analyzed. Can we actually say this was a purely qualitative study if it included quantitative approaches like the Likert scale?

**Response**: We used the Likert-style scale in Uganda to help participants focus their perspectives about “satisfaction” with MAP features. We used this tool to facilitate the debrief interview regarding features and whether they met participants’ needs, and to highlight priority areas for future refinements.

For clarity, we revised the Study design text to read as follows:

Also, we added a statement in Data Management and Analysis section to clarify:

**Results**

Line 261: spell out/define IFU.

**Response**: We have defined IFU in this line, per your request.

Line 272: what kind of challenges do MAPs address? Provide examples.

**Response**: Thank you for this question. We tried to address this in the Introduction section where we describe the potential value of MAPs for drug delivery (see paragraph 5 of the Introduction section), but we have added specific examples in these perceived benefits relative to existing HIV prevention and contraceptive methods in the Results section as well, per your request. The Introduction to this section now reads:

Were there any differences in perceptions and preferences between groups and countries?

**Response**: Thank you for this question. We did not find significant differences between countries or user groups. We saw strong similarities between user groups stated needs and preferences for HIV prevention and MPT products. We did find some nuances between groups, for example FSW and MSM, who already were aware of their risk of HIV and were more motivated to use HIV prevention, were more willing to accept MAP—even if it was larger than they preferred. They were more willing to accept a larger sized MAP especially if it could provide longer term protection. AGWY were not as willing to accept a large sized MAP. At this early stage of product development, when the active pharmaceutical ingredient is not yet identified, we felt the major findings of similarities were more relevant to report than the nuanced findings. **Our interpretation of these findings is already included in the Discussion section, paragraphs 2–7 (revised lines 628–691).**

**Discussion**

This section is missing reference to literature on current studies on other effective HIV prevention methods, e.g., vaginal ring, CAB-LA, etc. For instance, the authors do mention that it addresses issues seen in other HIV prevention methods, but it’s unclear what issues and in what way MAPs address those issues.

**Response**: We have added detail and citations to the Introduction and Discussions section to address the current state of oral PrEP introduction and other prevention options, such as dapivirine vaginal ring and long-acting injectable cabotegravir. Thank you.
